# Emotional consciousness preserved in patients with disorders of consciousness?

**DOI:** 10.1007/s10072-019-03848-w

**Published:** 2019-04-02

**Authors:** Jian Gao, Min Wu, Yuehao Wu, Ping Liu

**Affiliations:** 1 Department of Rehabilitation, Hangzhou Hospital of Zhejiang General Corps of Armed Police Forces, Hangzhou, 310051 China; 2 Department of Neurology & Brain Medical Centre, First Affiliated Hospital, School of Medicine, Zhejiang University, Hangzhou, 310003 China

**Keywords:** Emotional consciousness, Electroencephalography, Disorders of consciousness, Network connectivity

## Abstract

Increasing evidence from studies of brain responses to subject’s own name (SON) indicates that residual consciousness is preserved in patients with disorders of consciousness (DOC) and that specific network activation might provide evidence of consciousness. However, it remains unclear whether SON is suitable for detection of emotional consciousness; moreover, the particular aspects of brain network organization that are critical for consciousness are unknown. The present study used an innovative approach to explore affective consciousness in patients with DOC during emotional stimuli. EEG data were acquired from 15 patients and 15 healthy volunteers. We analyzed brain potentials and functional network connectivity with a passive emotional paradigm based on graph theoretical methods. Larger N1 or P3a was detected in patients upon exposure to emotional sound, relative to neutral stimuli. Brain topology revealed that emotional sound evoked significantly stronger network linkages in healthy controls; additionally, it evoked several connectivity changes in patients with DOC. In conclusion, emotional consciousness might be partially preserved in patients with DOC; moreover, EEG network patterns could provide new insights into the neural activity of emotional perception in these patients.

**Electronic supplementary material:**

The online version of this article (10.1007/s10072-019-03848-w) contains supplementary material, which is available to authorized users.

## Introduction

Probing consciousness in noncommunicating patients is an important challenge in the field of neuroscience. Thus far, the Coma Recovery Scale–Revised (CRS-R) continues to be a rational and practical choice for assessment of patients with disorders of consciousness (DOC) [1, 2]. However, because of motor and language deficits, the evaluation of non-reflex behavior is often both challenging and uncertain [3]. Notably, in these cases, a lack of responsiveness is not necessarily indicative of absence of consciousness and awareness. Recent publications have demonstrated preservation of awareness across the boundary of consciousness, as well as in patients with unresponsive wakefulness syndrome (UWS) [4, 5]. Yu et al. speculated that UWS preserved “affective consciousness” as evidenced by pain cries, which may indicate activation of the pain matrix (PM) [5]. By using an original rule extraction event-related potential (ERP) test, Faugeras et al. detected neural signatures of consciousness in patients who met clinical criteria for UWS [1]. Taken together, these findings suggest that patients with DOC exhibit preservation of unequivocal signs of consciousness. Additionally, these findings are indicative of the effectiveness of neurophysiological tools for covert residual consciousness detection in this specific patient population.

However, there is evidence to suggest that patients with DOC may not exhibit detectable cerebral responses at rest, or upon the application of simple brain stimulation paradigms, as such patients cannot cross the threshold for plasticity modifications; thus, they exhibit no detectable response [6]. Emotion is a key aspect involved in individuals’ experiences of their external environment and can persist in subjects with severe brain damage [7]. Emotional stimuli are likely to preferentially capture an individual’s attention and be processed by integration of primitive neural processes. Consequently, affective consciousness is the simplest and most fundamental variant of consciousness to study, regarded as “first-order consciousness,” which might persist in patients with DOC [5].

Previous neuroimaging studies have shown that DOC comprises a disconnection syndrome [8]. Reduced functional connectivity of the default mode network (DMN), frontoparietal network, and auditory network has been associated with impaired consciousness [8, 9]. Functional magnetic resonance imaging (fMRI) studies used block designs to localize responses to a diffuse network of brain regions, as fMRI relies upon the hemodynamic response, which can be followed on a per-second basis. Nevertheless, affective processes occur in the order of milliseconds; thus, neuroimaging data might lack the temporal resolution needed to capture the earliest emotional processes. However, electroencephalography (EEG), because of its millisecond-level resolution, is a particularly valuable method for measurement of rapid temporal brain dynamics.

In this study, we aimed to probe affective consciousness in patients with DOC using a new EEG-derived functional network analysis, and to identify a candidate marker to facilitate differential diagnosis of DOC.

## Experimental procedures

### Subjects

Fifteen patients with hypoxic-ischemic brain damage were recruited from the Department of Rehabilitation at Hangzhou Wujing Hospital. Of these 15 patients, seven met the diagnostic criteria for UWS; the remaining eight were diagnosed with minimally conscious state (MCS). All subjects met the following study inclusion criteria: (1) no centrally acting drugs, (2) no neuromuscular function blockers and no sedation within the 24 h prior to the study, (3) periods of spontaneous eye opening, and (4) with non-traumatic brain injury [10–12]. Demographic and clinical characteristics of the enrolled patients are shown in Table 1.

**Table 1:** General data comparison of DOC patients

	MCS	UWS	*P*
Age (years)	59 ± 15	53 ± 13	0.359
Gender (male/female)	5/3	4/3	0.833
Etiology (hemorrhage/anoxia)	7/1	6/1	0.919
Months since injury	4.2 ± 2.9	2.8 ± 2.1	0.292
CRS-R total scores	10 ± 4	5 ± 2	0.013

The study also recruited 15 age- and gender-matched healthy controls (HC). None of the controls had a history of brain injuries or neurological or psychiatric illnesses. Written informed consent was provided by the legal representative of each patient prior to the experiment. This study was approved by the Ethics Committee of the First Affiliated Hospital, School of Medicine, Zhejiang University and Hangzhou Wujing Hospital.

### Paradigm design

In the auditory oddball paradigm, four acoustic stimuli were produced at maximum 90-dB intensity; each was accompanied by an angry, happy, or neutral prosody. The standard stimulus (neutral voice) was a meaningless neutral sound (namely, the interjection “ah”), while the deviant stimulus (emotional voice) was the same sound with positive or negative affective prosody. These stimuli were chosen from a validated battery of vocal emotional expressions [13]. Trials proceeded as follows: first, a fixation-cross appeared in the center of the screen, followed by the sound stimuli after 1500 ms. Each sound sample had a duration of 700 ms, with an interstimulus interval of 1500 ms. The stimuli were presented to the patients in a block design; each block consisted of a total of 110 stimuli with 86 neutral standards, 12 happy deviants, and 12 angry deviants. All deviant sounds were presented in a randomly permuted order, ensuring that the same word was not presented in quick succession.

### EEG recordings and processing

EEG was performed using a 32-channel BrainCap (BrainAmp 32 MR, Brain Products GmbH, Munich, Germany) with the standard 10–20 system. All EEG electrodes were referenced online to FCz and re-referenced offline to the average of the left and right mastoids. A vertical electro-oculogram (EOG) was recorded supra-orbitally from the left eye, and a horizontal EOG was recorded from the right orbital rim. The impedance in all electrodes was maintained below 10 kΩ, and a 50-Hz notch filter was used. The EEG and EOG signals were amplified using a DC 1000-Hz bandpass filter and were continuously digitized at a sampling rate of 500 Hz.

EEGLAB was used for continuous EEG preprocessing. After offline referencing, the EEG signal was high-pass filtered at 0.1 Hz and subsequently low-pass filtered at 30 Hz. The EEG was then segmented into [− 200, 1000]-ms epochs. Artifact-free periods underwent independent component analysis (ICA) using the runica function; bad channels were interpolated using planar gradiometers incorporated in EEGLAB.

### Event-related potentials

To detect reliable differences with improved temporal and spatial resolution, massive univariate analysis was performed at each time point using parametric tests within the EEGLAB study framework. Multiple comparisons were corrected by the Benjamini and Yekutieli (2001) procedure, which ensured that the false discovery rate (FDR) would be < 5%. The regions with significant differences between conditions were marked with black bars at the bottom (*P* < 0.05, paired *t* test with 1000 permutations with FDR correction).

### Network connectivity

Phase locking value (PLV) was used to construct the corresponding brain networks. PLV is widely used for measurement of phase-synchronization among pairs of electrodes [14]; higher PLV value represents increased strength of phase-synchronization. The details of network construction and properties calculations, i.e., clustering coefficient (*C*), characteristic path length (*L*), global efficiency (*Ge*), and local efficiency (*Le*), are provided in the supplementary materials.

### Statistical analysis

Independent samples test and Fisher’s chi-square test were used to compare the continuous and categorical variables between MCS and UWS, respectively. Repeated measures analysis of variance (ANOVA) with *group* (HC, MCS, and UWS) as the between-subject factor and *condition* (neutral and emotional) as the within-subject factor was performed. When statistically significant differences were found, post hoc Bonferroni correction for multiple comparisons was conducted. When the interaction of *group* and *condition* was significant, simple effects tests were performed.

The sphericity assumption was assessed using Mauchly’s test prior to conducting repeated measures ANOVA. When the assumption was rejected, the Greenhouse-Geisser correction was used to adjust the degrees of freedom. Statistical analysis was performed using SPSS version 22.0 software.

## Results

### ERP results

**Fig. 1:**
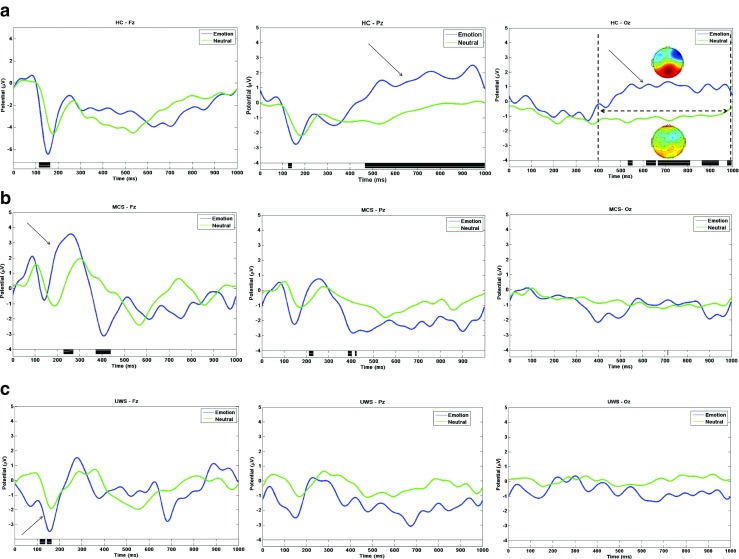
The ERP components underlying the presented emotional and neutral stimuli. Black bars in the bottom panel indicate regions of significant difference between conditions (*P* < 0.05, FDR correction). **a** The ERP waveforms at electrode Fz, Pz, and Oz in healthy controls. An obvious LPP was evoked by emotional sound. **b** ERP waveforms for MCS. A significant larger P3a shown at Fz. **c** ERP waveforms for UWS. Emotional sound evoked a significant larger N1 at Fz

Figure 1a shows the grand average ERPs at three midline electrode sites (Fz, Pz, and Oz), calculated for emotional and neutral stimuli in HCs. The N1 waveforms (with a negative dip between 100 and 200 ms) at Fz electrodes were more prominent for emotional stimuli. Figure 1a also shows the typical late positive potential (LPP) complex at the electrode Pz and Oz sites, ranging from 400 to 1000 ms with the stimulation of affective prosody in the HC group. Inspection of the scalp distribution of the waveform showed that the LPP was highest over central parietal-occipital sites (shown in top-right panel of Fig. 1).

However, only early auditory ERP components (e.g., N1, P3a) could be observed in either patients with UWS or those with MCS (Fig. 1b, c). An emotional effect was detected by P3a in patients with MCS, while the N1 effect was detected in patients with UWS. In contrast, no emotional evoked LPP was detected in the group with DOC.

### Network connectivity in neutral and emotional condition

**Fig. 2:**
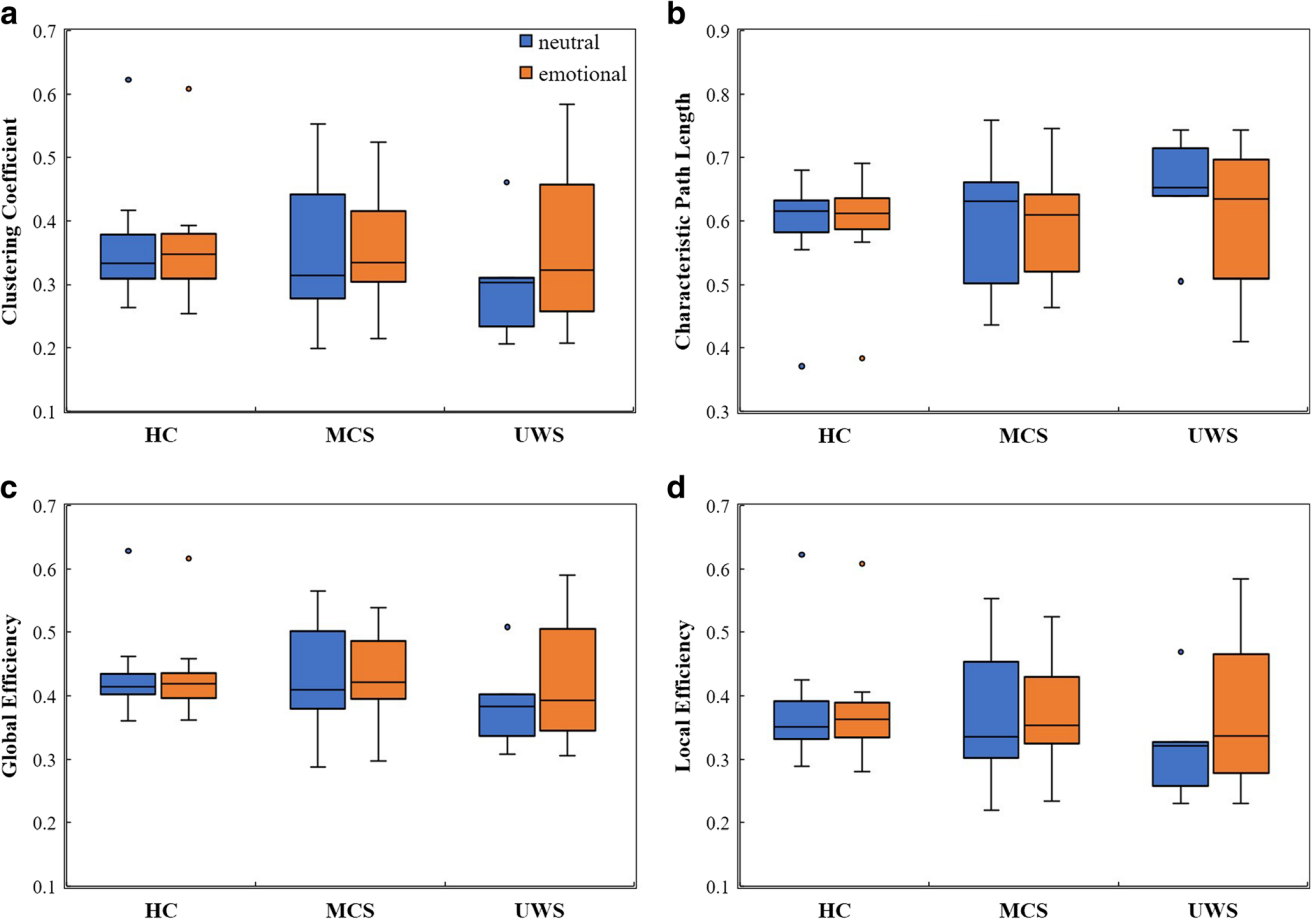
Boxplots of network properties estimated with neutral and emotional stimulation both in the healthy volunteer group (HC) and DOC groups. No emotional effect was detected in all three groups (repeated measures ANOVA*, P* > 0.05). And, the differences of network matrixes among hierarchical levels of consciousness were not significant (*P* > 0.05). (**a**) Clustering coefficient. (**b**) Characteristic path length. (**c**) Global efficiency. (**d**) Local efficiency

Regarding network properties, there were no significant differences in any of the network properties between neutral and emotional sound stimuli (Fig. 2, *P* > 0.05); repeated measures ANOVA revealed no significant main effect of the stimulation condition (shown in Table 2). Functional networks in both conditions had similar clustering coefficient, characteristic path length, global efficiency, and local efficiency values. Remarkably, individual differences in MCS and UWS groups were larger, as matrix data exhibited a discrete distribution. In contrast, in healthy volunteers, all network properties were more consistent (Fig. 2).

**Table 2:** Differences of network properties between neutral and emotional stimulation in three groups (HC, MCS, and UWS)

Network properties	Statistics	
	*F* values	*P* values
*C*		
Group	0.365	0.697
Stimulation	2.647	0.115
Group × stimulation	2.080	0.143
*L*		
Group	0.485	0.621
Stimulation	2.496	0.126
Group × stimulation	1.953	0.161
Ge		
Group	0.448	0.643
Stimulation	2.020	0.167
Group × stimulation	1.857	0.176
Le		
Group	0.387	0.683
Stimulation	2.503	0.125
Group × stimulation	2.033	0.150

*C* clustering coefficient, *L* characteristic path length, *Ge* global efficiency, *Le* local efficiency, *HC* healthy controls, *MCS* minimally conscious state, *UWS* unresponsive wakefulness syndrome

**Fig. 3:**
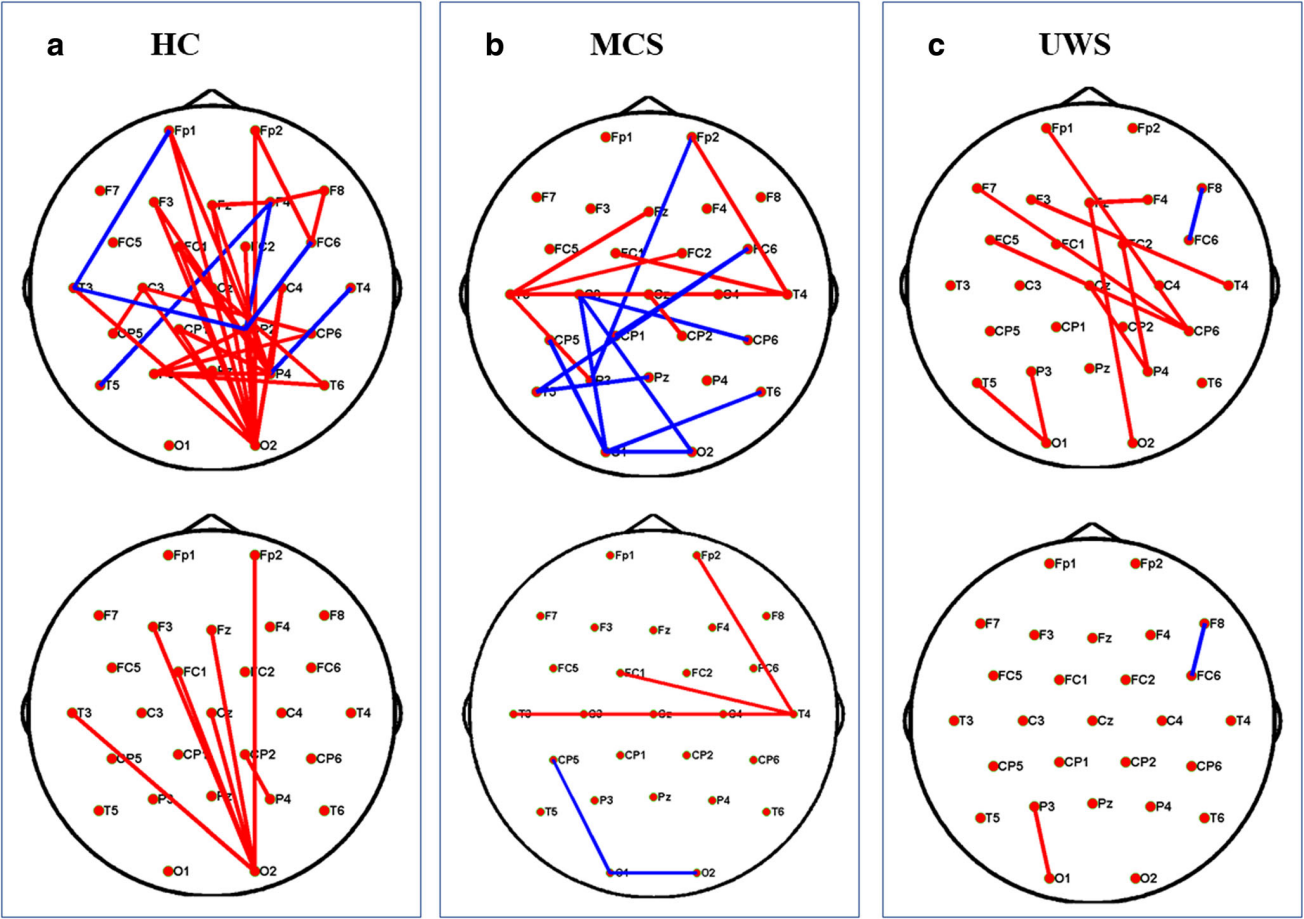
Functional connectivity in three groups for neutral and emotional auditory stimulation. **a**–**c** mean comparisons of brain connectivity with different auditory conditions in healthy controls, MCS, and UWS, respectively. The red lines denote significantly increased linkage strength with emotional stimulation than with neutral stimulation, while the blue lines denote significantly decreased linkage strength (*P* < 0.05, uncorrected, on the upper; *P* < 0.05, Bonferroni corrected, at the bottom). (Color figure online)

In the next step, we further explored brain topology differences in the two contrasting conditions. As shown in Fig. 3a, emotional sound evoked significantly stronger network linkages in HCs (*P* < 0.05), particularly with regard to connections between frontal-occipital and parietal-occipital lobes. In patients with MCS, there were several increased linkages in temporal areas and decreased linkages in the occipital cortex (Fig. 3b); patients with UWS also showed increased information flow in temporal lobes (*P* < 0.05, uncorrected). Nonetheless, an improved network connection was not present after Bonferroni correction (Fig. 3c). Moreover, both healthy subjects and patients with MCS exhibited multiple network connection changes with both increased and decreased linkages, while alternation in patients with UWS was simpler: Only irregularly increased activation was detected, as shown in the top panel of Fig. 3c.

### Network connectivity among levels of consciousness

**Fig. 4:**
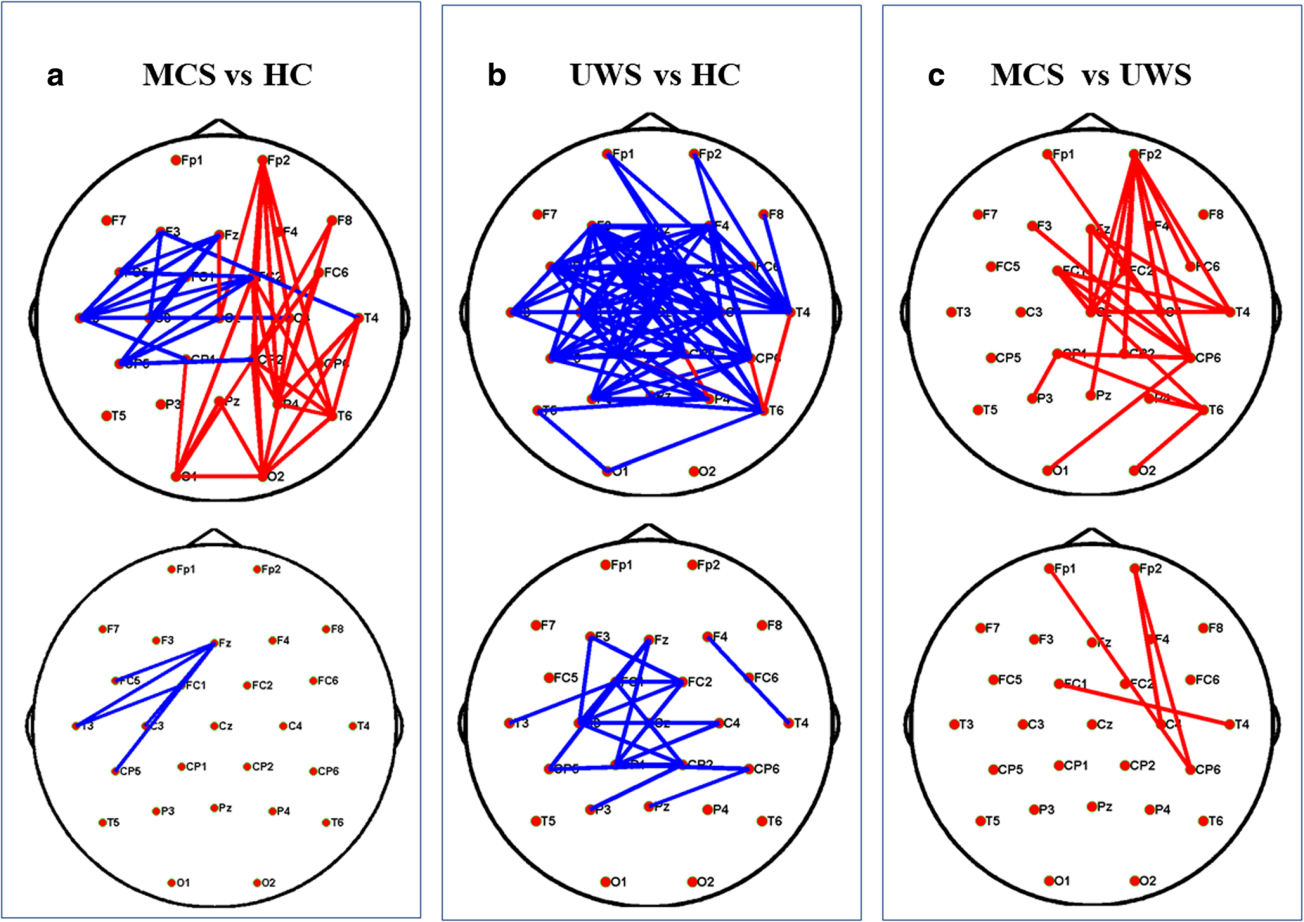
Functional connectivity comparisons among the levels of consciousness underlying neutral sound stimulation. **a** Differences of network connectivity between MCS and healthy controls. **b** Significantly decreased network connectivity in UWS than in the healthy volunteers. **c** The network difference between MCS and UWS. Red lines mean significantly increased connectivity, and blue lines mean significant decreased connectivity (*P* < 0.05, uncorrected, on the upper; *P* < 0.05, Bonferroni corrected, at the bottom). (Color figure online)

**Fig. 5:**
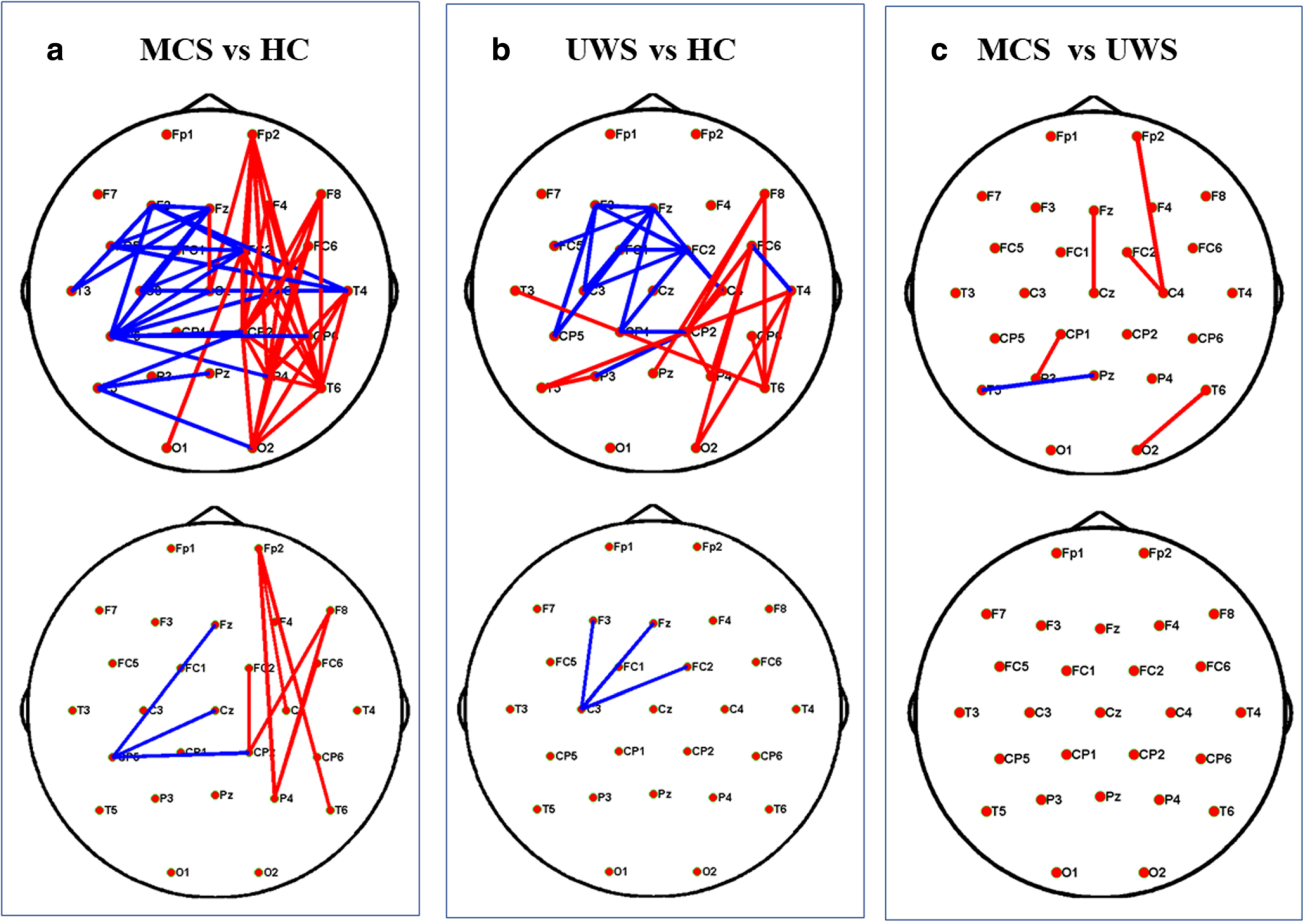
Spatial topology differences of the PLV network among the levels of consciousness underlying emotional sound stimulation. **a** Differences of network connectivity between MCS and healthy controls. **b** Network connectivity difference between UWS and healthy volunteers. **c** The subtle brain network difference between MCS and UWS. Red lines denote significantly increased linkage strength, while the blue lines denote significantly decreased linkage strength (*P* < 0.05, uncorrected, on the upper; *P* < 0.05, Bonferroni corrected, at the bottom). (Color figure online)

Surprisingly, all network properties showed no significant differences among the levels of consciousness, since there was no significant main effect of group, nor an interaction between group and stimulation condition (Table 2). Functional networks in all three groups also showed similar global efficiency and clustering coefficient values (Fig. 2). Hence, all showed conservation of global properties of small-world networks [15]. Thus, despite marked differences in states of consciousness between patients with DOC and healthy subjects, their brain networks showed conservation of global properties of small-worldness. Finally, by performing further spatial information analysis, we detected brain topology differences among the three groups and described detailed network connectivity distinctions, both in neutral and emotional settings. Compared with HCs, patients with MCS frequently showed abnormally reduced hubness of nodes in the frontal-parietal cortex and abnormally increased hubness of nodes in the frontal-occipital and temporal-occipital cortexes (Figs. 4a and 5a). Figures 4b and 5b depict distinct differentiated network linkages between patients with UWS and HCs for neutral and emotional conditions, respectively. Patients with UWS exhibited widespread impaired cortical connectivity; however, when these patients were exposed to emotional acoustic stimulation, the gradual reduction in connectivity was significantly relieved (Fig. 5b). In a comparison between patients with MCS and those with UWS, the patients with UWS showed impaired connectivity, particularly in the right frontal-parietal cerebral cortex (Fig. 4c); surprisingly, this difference diminished with emotional stimulation (Fig. 5c).

## Discussion

A considerable amount of recent evidence indicates that some patients with DOC might show preservation of covert awareness, detectable with fMRI and EEG. These findings are illuminating and support further studies into neural mechanisms regarding the existence of consciousness in such patients. However, to minimize semantic processing, emotion was portrayed through meaningless interjection in our study; further, neural mechanisms were explored by conventional EPR and functional network connectivity.

LPP has been shown to be specifically sensitive to the regulation of emotional responses [16]. In the current study, the typical LPP in the HCs demonstrated existing emotional sound processing and served as an index of cognitive demands, representing allocation of attention resources, as well as an index of downstream processes resulting from increased activation of amygdala linkage to memory encoding and storage [16, 17]. Hence, it seems reasonable to speculate that emotional regulation and memory encoding are greatly attenuated in patients with DOC. However, an intriguing phenomenon was that of prominent frontal P3a in patients with MCS, including a larger amplitude underlying emotional stimulation. Hence, involuntary attentional orientation might be preserved in patients with MCS, since P3a has been suggested to serve as a biomarker of exogenous attention [18]. Discrepant N1 between neutral and emotional conditions in patients with UWS merely suggested the existence of early automatic sensory identification for patients with DOC, as N1 indexes pre-emotional perception of physical parameters [19].

Next, we focus on discussion of functional network connectivity regarding emotion processing in all subjects. All network properties failed to reveal emotional effects, both in HCs and in patients with DOC. Nevertheless, spatial topology visually represented contrasting network connectivity, for several reasons addressed below. Network properties, which serve as direct statistical descriptions of network connectivity, result from average calculations of both increased and decreased functional connectivity across all parts of the brain; thus, they might fail to encompass all information content related to the network [20]. However, the complete spatial information of a network is considerably more complex than its representative statistical measurements.

Consistent with ERP findings that emotional stimuli increased the amplitude of the specific ERP components (LPP) linked to stimulus salience, the brain topology of healthy people has shown sustained increased information flow [16]. Generally, LPP comprises a broad parietal-occipital positivity [21]. Collectively, in our study, emotional stimuli prompted prominent network connectivity in parietal-occipital lobes. In addition, imaging studies of cross-modal stimuli revealed that affective sound processing might lead to activation of the visual cortex in the occipital area [22, 23]; these results coincided with our finding that remarkable activation in the occipital lobe was evoked by emotionally deviant sound.

For patients with DOC, only slight temporal activation was detected. Although these positive findings might demonstrate the ability of a brain to discriminate the presence of a given target feature, such as the affective tone in our study, it remains unclear whether emotional conscious experience can be detected in patients with DOC. Current theory states that the frontoparietal network is critical for conscious perception [24]; however, Demertzi et al. highlighted the contribution of temporal auditory cortex to the level of consciousness [25]. Thus, it may be reasonable to speculate on the existence of signs of consciousness in patients with MCS: evidence from increased functional network connectivity. Additionally, a further validation study with larger samples should be conducted.

Third, we discuss the significant gain in discrimination obtained by network comparison among hierarchical levels of consciousness. Our results concurred with and complemented previous fMRI studies [15], which demonstrated that brain network properties were unlikely to be useful biomarkers for stratified levels of consciousness. In a study by Sinitsyn, only subtle differences were captured between UWS and MCS in a whole-brain analysis [9]. Moreover, Achard indicated that global network properties of functional connection were homeostatically conserved in comatose patients and showed no significant differences from properties of HCs [15]. Additionally, the present study demonstrated conservation of fundamental network properties, such as small-worldness, across a wide range of clinical cases of DOC. Indeed, additional data have confirmed the existence of “small-world” properties in most clinical disorders [26, 27]. This suggests that global topological properties are insufficient to describe the brain network organization required for normal consciousness [28, 29].

However, when we separately examined network topology analysis in emotional and neutral situations, we found evidence of highly significant abnormalities in patients with DOC. First, in patients with MCS, abnormally increased activation in the occipital lobe could be ascribed to the loss of a top-down (frontoparietal network) inhibitory gating mechanism [30]. Regarding connectivity in patients with UWS, our report was in accordance with prior findings, which suggested that UWS was associated with a massive disruption of complex brain functional networks [31, 32]. Another notable finding was obvious right frontoparietal network activation in patients with MCS, compared with those with UWS, during neutral sound stimulation. Current reports indicate that frontoparietal activation is present in subliminal stimulus processing and is associated with high-level cortical perception [33]. Hence, disruption of top-down processes of higher-order associative cortices is implicated in consciousness and may clearly differentiate UWS from MCS.

Strikingly, the contrast between patients with MCS and those with UWS disappeared upon emotional stimulation. Two hypotheses may explain these surprising findings. First, the emotional target was regarded as an infrequent stimulus and required increased attention allocation [16]. HCs, with normally functioning feedback and regulation systems, may have attenuated the allocation of attention and awareness with regard to deviant stimuli [34]. Since the deviant sound always followed neutral prosody, it was predictable. Similarly, patients with MCS also exhibited partial preservation of feedback processing [33]. Moreover, there was additional evidence from network changes induced by pleasant or unpleasant musical stimuli. A subset of UWS patients showed increased connectivity during the unpleasant stimulus, while healthy subjects exhibited no changes [35]; nevertheless, the neural mechanisms remained elusive.

To the best of our knowledge, there have been few studies regarding whether etiological heterogeneity contributed to differences in brain responses to emotionally laden auditory stimuli; in the present study, we considered etiology and only enrolled nontraumatic patients. A voxel-based morphometry study compared structural aspects between traumatic and non-traumatic brain injury; notably, patients with traumatic brain injury exhibited wider network injury, but no structural differences were observed between patients with UWS and those with MCS [16]. We hypothesize that the difference in emotion-induced brain activation is related to the etiology of the brain injury. However, our study involved a limited number of patients; hence, further studies are needed to better characterize these differences.

Comparatively speaking, there were limitations in our study. The diversity of injury sites might confound the results. Nonetheless, to avoid the intense mechanical damage involved in traumatic brain injury, our study solely involved patients with non-traumatic DOC. Another limitation is the limited sample size; however, it may still be illuminating as a starting point to probe emotional consciousness with EEG network analysis, and further studies with larger samples are needed in the next phase. Moreover, pleasant and unpleasant conditions were not analyzed separately, although there is increasing evidence that cerebral responses to positive and negative affective valence may be unequal. Our present study provides a preliminary characterization of emotional processing ability in patients with DOC, without valence discrimination. Therefore, further studies are likely to more deeply explore differences among various valence stimulations.

## Electronic supplementary material

ESM 1 (DOCX 25 kb)
