# Supplementary material for: Emotional consciousness preserved in patients with disorders of consciousness?
Source: Neurol Sci. 2019 Apr 2;40(7):1409–18. doi: 10.1007/s10072-019-03848-w (PMC6579782; doi:10.1007/s10072-019-03848-w)
Supplement: Supplementary file 1 — (DOCX 25 kb) [file 10072_2019_3848_MOESM1_ESM.docx]

**Methods:**

Let *t* denote the sampling period and *N* denote the sample number, for two time series, *x(t)* and *y(t)*, the instantaneous phases are *ɸ_x_(t)* and *ɸ_y_(t)*, respectively, and the PLV is formulated as,

**** (1)

For each subject, the final weighted brain network, an adjacency matrix was obtained by averaging matrices of those artifact-free data segments. Finally, based on all these constructed brain networks, the independent *t*-test was used to probe the differences of brain architecture between two groups for both the standard and deviant datasets. The results of all statistical tests were corrected for multiple comparison using Bonferroni correction.

Here, graph theoretical analysis was performed on the phase synchronization matrices and we subsequently measured the network efficiency of emotional processing with network properties. Let *w_ij_* and *d_ij_* represent the PLV value and shortest path length between nodes *i*-th and *j*-th, respectively, *n* represents the node number, and *Ψ* represents the set of nodes. Then, the brain connectivity toolbox was used to calculate weighted network properties, i.e., clustering coefficient (*C*), characteristic path length (*L*), global efficiency (*Ge*), and local efficiency (*Le*). The definitions of four referred network properties were formulated as

**$C=\frac{1}{n}\sum_{i\in\Psi} \frac{\sum_{j,h\in\Psi} \left(w_{ij}w_{ih}w_{jh}\right)^{1/3}}{\sum_{j\in\Psi} w_{ij}\left(\sum_{j\in\Psi} w_{ij}-1\right)}$** (2)

**$L=\frac{1}{n}\sum_{i\in\Psi} \frac{\sum_{j\in\Psi,j\neq i} d_{ij}}{n-1}$** (3)

**$Ge=\frac{1}{n}\sum_{i\in\Psi} \frac{\sum_{j\in\Psi,j\neq i} \left(d_{ij}\right)^{-1}}{n-1}$** (4)

**$Le=\frac{1}{n}\sum_{i\in\Psi} \frac{\sum_{j,h\in\Psi,j\neq i} \left(w_{ij}w_{ih}\left[d_{jh}\left(N_{i}\right)\right]^{-1}\right)^{1/3}}{\sum_{j\in\Psi} w_{ij}\left(\sum_{j\in\Psi} w_{ij}-1\right)}$** (5)
